# COVID-19 and Sepsis in an Atypical Case of Mixed Connective Tissue Disorder Presenting With a Myasthenic Crisis

**DOI:** 10.7759/cureus.29092

**Published:** 2022-09-12

**Authors:** Lav Kumar Shah, Biswas Pant, Nashruva Mony, Sumitanand Mishra, Januka Gaire, Sandesh Sharma

**Affiliations:** 1 Surgery, Patan Academy of Health Sciences, Lalitpur, NPL; 2 Medicine, Patan Academy of Health Sciences, Lalitpur, NPL; 3 Medicine, Nepal Korea Friendship Municipality Hospital, Bhaktapur, NPL; 4 Radiology, Himal Hospital, Kathmandu, NPL; 5 Internal Medicine, Nepal Bal Tatha Mahila Prasuti Sewa Polyclinic Pvt. Ltd., Birendranagar, NPL; 6 Internal Medicine, Kaushallya Memorial Hospital Pvt. Ltd., Banke, NPL

**Keywords:** systemic scleroderma, acute polymyositis, systemic lupus erythema, neuromuscular junction, covid-19, myasthenic crisis, mixed connective tissue disease

## Abstract

Myasthenia gravis (MG) is an autoimmune illness that causes neuromuscular junctions to be damaged by anti-acetylcholine receptor antibodies. It is a very rare condition that is more common among women. Fatigable fluctuating diplopia or ptosis is the characteristic early appearance of this condition. Dysphagia or dysphonia may be present in rare cases. This illness can affect any group of skeletal muscles, including those in the neck and upper limbs. It can also affect the muscles that help you breathe, which can lead to breathing failure.

We present a case of a 20-year-old female diagnosed with mixed connective tissue disease presenting with acute respiratory failure as the initial presentation of MG. Clinicians have to have a high index of suspicion for myasthenia when patients arrive with fatigable muscle weakness. This will cut down on the amount of money spent on investigations and the risk of morbidity.

## Introduction

Mixed connective tissue disease (MCTD) is diagnosed by the presence of overlapping features of three distinct autoimmune diseases, which include systemic lupus erythematosus (SLE), polymyositis, and systemic sclerosis, at the same time. MCTD is most commonly presented with features suggestive of synovitis and edema of the hands, which are associated with features of Raynaud's phenomenon. The patients typically present with muscle pain and weakness of their muscles. The majority of patients have anti-ribonucleoprotein (RNP) antibodies, which are also present in SLE without the overlap syndrome. The term "overlap syndromes" is used to describe a large and varied group of conditions that share symptoms with more than one well-defined rheumatic disorder. Clinical manifestations can occur in a variety of organ systems and typically appear sub-acutely. The organs that are affected match the main symptoms of the different rheumatic diseases [1]. The presence of anti-U1 RNP is a significant finding for MCTD diagnosis. Myasthenia gravis (MG) is a kind of autoimmunity that can occur sporadically and is characterized by decreased neuromuscular transmission. The extraocular muscles are often the target of anticholinergic autoantibodies, which leads to varying muscular fatigability as well as bilateral diplopia and ptosis, both of which are typically worse in the evening. It is one of the most prevalent reasons for respiratory discomfort that is caused by neurological conditions. In approximately 21% of cases, the onset is at the age of 60, while bulbar or respiratory muscle weakness occurs in approximately 30% of cases. Myasthenia crisis (MC) affects approximately 15%-20% of people with MG [2]. These patients may present with acute respiratory failure that necessitates intubation if the initial diagnosis of MG or any condition such as respiratory infection, emotional stress, surgery, pregnancy, or any other type of stress in which MC can be observed in MG patients is delayed due to lack of proper clinical protocol. Both of these scenarios increase the likelihood that the initial diagnosis will be delayed.

## Case presentation

A 20-year-old female presented with a rash and tightening of her skin that had been going on for two to three months, pain and swelling in her upper and lower limb joints for one month, and trouble in swallowing. She was evaluated as a medical outpatient and admitted to the medical ward for further evaluation. The patient had these above-mentioned symptoms since two years, which gradually progressed over the course of time. During the past two years, the patient used to have occasional shortness of breath, which progressed over time and developed into exercise intolerance for which she had to seek medical attention in her hometown. The other symptom that made her seek medical attention and visit a primary care physician was pain and swelling in the small joints of her upper and lower limbs. The patient was evaluated clinically and was advised to undergo lab tests, which revealed anemia (low hemoglobin). As the patient belonged to a specific community where the prevalence of sickle cell disease (SCD) is high, the patient was suspected to have SCD and was evaluated for that and other possible causes of anemia. She was managed symptomatically with iron, calcium, and painkillers and was discharged with an advice to follow up with reports. Her lab tests failed to reveal SCD and other causes of anemia. Hence, for the next 18-24 months, the patient was managed symptomatically without any definitive diagnosis. However, her symptoms progressed significantly and with increasing severity, following which she was referred to a higher treatment center for further evaluation and management.

The patient was evaluated and provisionally diagnosed with inflammatory myositis, autoimmune hepatitis, and hypothyroidism based on her clinical presentation and initial lab results. However, over the course of her stay, she developed a spectrum of symptoms such as altered mentation, irrelevant talk, drowsiness, and a low Glasgow Coma Scale (GCS) score. She was intubated with the help of propofol and remifentanil combination. She was admitted to the intensive care unit (ICU) for further management. After nine days of intubation, on January 16, 2022, due to improvement in her symptoms on clinical evaluation and after all the criteria for extubation were met, an extubation trial was done and the patient was extubated on January 16, 2022. However, the patient was unable to tolerate it and developed gradual difficulty in breathing, for which she was re-intubated on the same day. Over the course of her ICU stay, the patient was evaluated and examined daily, which revealed persistently decreased muscle power of 2 out of 5 in her bilateral upper limbs and lower limbs, including orbicularis oculi. Following these findings, an ice pack test was performed, which failed to reveal any significant improvement. However, before ruling out critical illness myopathy due to a high degree of clinical suspicion, an acetylcholine receptor antibody (AchR Ab) test was done to rule out myasthenia gravis. The patient's AchR Ab was positive (0.58 mmol/l), which was significantly above the baseline of 0.40. Subsequently, her anti-nucleic acid antibody (ANA) profile was also positive for anti-U1 RNP. The patient was diagnosed with MCTD with myasthenia gravis, hypothyroidism, and anemia of chronic disease. High-resolution computed tomography (HRCT) was done to rule out thymoma. The patient was managed with a high dose of anticholinesterase pyridostigmine (90 mg every three hours) and prednisolone (60 mg), which was gradually tapered to 10 mg. She underwent tracheostomy on February 4, 2022. During her course in the ICU, she developed a persistent fever and hypotension.

A chest X-ray was done, which revealed consolidation, following which bronchoscopy and bronchoalveolar lavage (BAL) were done and the sample was sent for culture and sensitivity, Gram stain, acid-fast bacillus (AFB) stain, and gene X-pert. Her blood and urine samples were also sent for culture and sensitivity testing. Her blood and BAL cultures revealed growth of Acinetobacter and urine cultures revealed fungal growth; a diagnosis of ventilator-associated pneumonia (VAP) and urinary tract infection (UTI) was made. She was treated according to sensitivity results, and her fever subsided after two days of antimicrobial treatment. However, her muscle power failed to improve, and plasmapheresis/intravenous immunoglobulin (IVIG) was planned for her. Due to cost issues and unavailability of resources immediately, the patient was switched to a high dose of prednisolone (60 mg). The patient's muscle power improved slightly, and she was kept on the same treatment. The patient had gradual and slow improvement in her muscle power, and it was planned to wean off ventilator support when she was screened for severe acute respiratory syndrome coronavirus disease 2019 (SARS COVID-19). Due to the outbreak of the third wave in our setting, the patient tested positive for SARS COVID-19. She was shifted to the COVID ICU. Her stay in the COVID ICU was uneventful and she was transferred back to the medical ICU once her polymerase chain reaction (PCR) report was negative after 11 days.

After the patient was transferred back to the medical ICU, she was evaluated clinically and physical examinations were performed, which revealed marked improvement in muscle power to grade 4. The patient was gradually and successfully weaned off ventilator support. The patient was scheduled for the removal of the tracheostomy tube. After a week of successful partial and complete tracheostomy tube occlusion trials, the tracheostomy tube was successfully removed on March 14, 2022, around the 65th day of the ICU stay. The patient was kept under 24-hour observation without any issues and was shifted out to the medical ward after 66 days of the ICU stay. The patient was discharged 72 days after hospital admission with an advice to follow up in seven days. The patient was also counseled about the possible need for elective thymectomy in the future.

## Discussion

A myasthenic crisis is a potentially a lethal event in MG characterized by a worsening of myasthenic weakness and the need for mechanical or noninvasive ventilator assistance [3]. Although the respiratory muscles are weak, considerable bulbar (oropharyngeal) muscle weakness frequently coexists with the respiratory muscle weakness and, in rare instances, may be the main hallmark of respiratory failure. If this causes an upper airway blockage or severe dysphagia with aspiration, the person needs to be intubated to help him/her breathe.

The initial diagnosis of mixed connective disorder puts the physician in a dilemma because MCTD has a wide range of presentation involving most of the systems. Even more confusion is added by the overlap syndrome, which consists of a varied group of conditions that share symptoms with more than one well-defined rheumatic disorder. Clinical manifestations can occur in a variety of organ systems and typically appear sub-acutely. The organs that are affected correspond to the main symptoms of the various rheumatic diseases. Initially, our patient presented with a respiratory problem and joint pain, and the physician thought the patient had a flair of SLE, which is a component of MCTD. After being admitted to the hospital, she also got an infection from the hospital, pneumonia from the ventilator, and a COVID-19 infection. The muscle weakness, which she developed over a period of time, was the turning point in her MC diagnosis, which was confirmed by laboratory tests as mentioned in the case presentation.

It is estimated that 2%-3% of MG patients experience a myasthenic crisis each year [4]. Myasthenic crises are the first indication of myasthenia gravis in 13%-20% of people [5]. Myasthenia gravis is frequently diagnosed in the first few years after an MG diagnosis, when the condition is usually in its most active state. The illness has a bimodal distribution; it is seen in women most commonly between the second and third decades of life, while it is most common in men between fourth and eighth decades. It expresses itself most frequently as a skeletal muscular weakness, especially affecting the extraocular muscles and, to a lesser extent, the muscles of mastication. Symptoms of this condition might vary from person to person. Dysphagia has been recorded as a symptom of myasthenia in older males, with the condition rapidly progressing to respiratory failure [6]. Aside from the usual muscles that are affected, this condition could affect any group of muscles, including the muscles of the upper limb and the neck.

Many reasons can bring on a myasthenic crisis, but an infection is the most common simultaneous cause [7,8]. It is also possible to experience this after undergoing surgery (such as a thymectomy), becoming pregnant, giving birth, or discontinuing the use of immunotherapeutic drugs [9]. On top of that, a myasthenic crisis is a normal part of how myasthenia gravis gets worse over time.

Numerous medications have the potential to exacerbate the strength loss associated with myasthenia [10]. Magnesium, procainamide, beta-blockers, quinidine, and some antibiotics (such as aminoglycosides, fluoroquinolones such as ciprofloxacin and levofloxacin, erythromycin, and azithromycin) can increase the likelihood of this happening.

People with stable myasthenia gravis are usually given anticholinesterases that work in an indirect manner, such as pyridostigmine and neostigmine. A cholinergic crisis is a serious side effect that can happen if you take too much anticholinesterase, and then it may become hard to tell the difference between that and myasthenia gravis getting worse. When anticholinesterase medicines are used, they can cause sudden weakness. This is called a "cholinergic crisis." But cholinergic crises are very rare if the amount of pyridostigmine taken is kept at less than 120 mg every three hours. Given how rare a cholinergic crisis is, it's unlikely that growing weakness is caused by an cholinergic overdose, unless it's clear that the amounts being taken are much higher than stated. If not, it is reasonable to think that the patient's myasthenia gravis is getting worse, even though cholinergic side effects are happening, and to start treating the patient accordingly.

Impending myasthenic crisis is described as a rapid clinical worsening of MG that, in the treating clinician's opinion, could escalate to crisis in the short term (days to weeks) for patients who have already been diagnosed with the disease [11]. A "myasthenic crisis" is when the condition gets so bad that mechanical breathing, intubation, or both are needed because the respiratory and/or bulbar muscles have become too weak. Up to 20% of MG cases begin with a myasthenic crisis, and the underlying reason for neuromuscular respiratory failure is frequently unknown at the time of diagnosis [12]. Whenever possible, immunologic or electrophysiologic testing should be done to confirm myasthenia gravis in these circumstances.

The clinical presentation of SARS-CoV-2 infection in patients can vary widely, from asymptomatic infection to severe disease. Certain preexisting conditions increase a patient's risk of developing severe COVID-19. Some examples of these conditions and their associations are as follows: being over the age of 65, being diagnosed with cancer, being diagnosed with cardiovascular disease, being diagnosed with chronic kidney disease, being diagnosed with chronic liver disease, being diagnosed with diabetes, being diagnosed with advanced or untreated HIV infection, being pregnant, being a smoker, receiving a transplant, and receiving immunosuppressive therapy [13].

The clinical recovery of such patients requires close monitoring by medical staff. Clinically managing a patient with COVID-19 effectively requires addressing not only the underlying medical condition that necessitated ICU admission but also any coexisting comorbidities and nosocomial complications. Also, due to COVID-19 infection, an increase in morbidity and mortality has been observed [14].

## Conclusions

Myasthenia gravis should be suspected in young female patients who present with abrupt respiratory failure, even if there are no typical symptoms present, as in this patient. Our patient had the typical feature of respiratory failure and most of the initial evaluation was going on in the line of mixed connective tissue disorder with respiratory infection. However, she had myasthenia gravis, which acutely presented as a myasthenia crisis due to superinfection. It is essential to recognize myasthenia in its early phases so that proper treatment may be administered. While examining MCTD, a doctor might miss a myasthenia crisis once in a while. The overall hospital stay duration is also increased due to the COVID-19 infection in patients, along with an increased risk of morbidity. Hence, clinicians need to have a high index of suspicion for this condition.
